# Non-coding RNAs derived from an alternatively spliced REST transcript (*REST-003*) regulate breast cancer invasiveness

**DOI:** 10.1038/srep11207

**Published:** 2015-06-08

**Authors:** Nan Sook Lee, Oleg V. Evgrafov, Tade Souaiaia, Adrineh Bonyad, Jennifer Herstein, Joo Yeun Lee, Jihong Kim, Yan Ning, Marcos Sixto, Andrew C. Weitz, Heinz-Josef Lenz, Kai Wang, James A. Knowles, Michael F. Press, Paul M. Salvaterra, K. Kirk Shung, Robert H. Chow

**Affiliations:** 1 Physiology & Biophysics and Zilkha Neurogenetic Institute, University of Southern California, Los Angeles; 2 Dept of Biomedical Engineering, University of Southern California, Los Angeles; 3 Psychiatry & the Behavioral Sciences, University of Southern California, Los Angeles; 4 Neuroscience Graduate Program, University of Southern California, Los Angeles; 5 Dept of Medicine, Norris Cancer Center, University of Southern California, Los Angeles; 6 Pasadena City College, Pasadena; 7 Dept of Ophthalmology, University of Southern California, Los Angeles, CA; 8 Dept of Pathology, Norris Cancer Center, University of Southern California, Los Angeles; 9 Department of Neuroscience, Beckman Research Institute of the City of Hope, Duarte, CA.

## Abstract

RE1-Silencing Transcription factor (REST) has a well-established role in regulating transcription of genes important for neuronal development. Its role in cancer, though significant, is less well understood. We show that REST downregulation in weakly invasive MCF-7 breast cancer cells converts them to a more invasive phenotype, while REST overexpression in highly invasive MDA-MB-231 cells suppresses invasiveness. Surprisingly, the mechanism responsible for these phenotypic changes does not depend directly on the transcriptional function of REST protein. Instead, it is driven by previously unstudied mid-size (30–200 nt) non-coding RNAs (ncRNAs) derived from the first exon of an alternatively spliced REST transcript: *REST-003*. We show that processing of *REST-003* into ncRNAs is controlled by an uncharacterized serine/arginine repeat-related protein, SRRM3. SRRM3 expression may be under REST-mediated transcriptional control, as it increases following REST downregulation. The SRRM3-dependent regulation of *REST-003* processing into ncRNAs has many similarities to recently described promoter-associated small RNA-like processes. Targeting ncRNAs that control invasiveness could lead to new therapeutic approaches to limit breast cancer metastasis.

One of the most important events in the natural history of breast cancer is the transformation of intraductal carcinoma to invasive disease^1^. If left untreated, invasive cells can progress to widespread metastases associated with a worsening prognosis. A major goal of cancer research is to identify the underlying mechanisms that regulate the *in situ* to invasive transition, in order to support new therapeutic strategies that would benefit cancer patients.

Recent work establishes a strong correlation between cancer invasiveness and the loss of RE1-Silencing Transcription factor (REST), a well-characterized protein best known for suppressing neuronal genes during development^2^,^3^,^4^,^5^,^6^,^7^. REST activity is suppressed during neurogenesis partly by reduced expression^6^,^7^ as well as by ubiquitin-induced proteolytic degradation^8^. Suppression or loss of REST function may also result from alternative splicing. An alternatively spliced form of REST, called REST4, has been identified in several cancer types that do not express normal REST transcripts (e.g., neuroblastoma^9^, small cell lung cancer^5^,^10^, and breast cancer^4^). In neurons, alternative splicing of REST into REST4 is controlled by a neuronal-specific Ser/Arg repeat protein (nSR100/SRRM4). Interestingly, REST can directly silence SRRM4 expression, thereby preventing alternative splicing and thus acting as an on/off switch for neuronal gene expression^11^,^12^. A similar REST-dependent alternative splicing mechanism could conceivably play a role in acquisition of neuronal-like properties of breast cancer cells, enabling them to become invasive^13^.

In addition to REST4 splicing, extensive alternative splicing of REST pre-mRNA has been detected in cancer tissues and cell lines^14^. At least 45 variants have been identified, some of which may code for modified proteins and others that are non-coding RNAs (ncRNAs). Significant generation of short ncRNAs in proximity to transcriptional start sites (TSSs) from human cells has recently been described^15^. An intriguing example is the promoter-associated short non-coding RNAs (PASRs) that appear to be derived from the 5’ ends of known protein-coding genes^15^,^16^,^17^,^18^. PASRs are generally in the size class of less than 200 nt but longer than typical inhibitory RNAs (i.e., >30 nt)^19^.

Here, we used RNA deep sequencing to identify a previously uncharacterized alternatively spliced form of the REST gene, *REST-003*, that plays a key role in controlling cancer cell invasiveness. *REST-003* is a non-coding RNA that is processed into a complicated series of sense and antisense ncRNAs with sizes ranging from 30–200 nt. The alternative splicing of REST transcript in invasive cells appears to be controlled by a previously unstudied SR protein, SRRM3, as well as by the REST protein itself. REST expression results in downregulation of both SRRM3 and *REST-003* transcript. *REST-003* levels correlate positively with cancer cell invasiveness, and knockdown of *REST-003* is sufficient to convert invasive cancer cells to a non-invasive phenotype. Our results reveal regulatory interactions among REST, *REST-003*, and a cancer cell-specific splicing activator (SRRM3) that may coordinate gene regulation required for development of the invasive cancer phenotype in breast cancer.

## Results

### REST levels correlate negatively with cancer invasion in breast cancer cell lines

We explored the REST-dependent invasive phenotype in more detail using two breast cancer cell lines: MDA-MB-231, which is strongly invasive, and MCF-7, which is weakly invasive. We first confirmed the invasive potential of each cell line using Matrigel invasion chamber assays^20^ (Fig. S1A). In agreement with published studies^2^,^3^, REST mRNA and protein levels were higher in MCF-7 relative to MDA-MB-231 cells (Fig. S1B, C).

We next downregulated *REST* in MCF-7 cells using two small interfering RNAs (siRNAs) (si-REST_1 and si-REST_2; see Fig. S1D and Table S1). Treated cells exhibited increased invasiveness in Matrigel assays (Fig. S1E). We then overexpressed wild-type (wt) REST^21^ in MDA-MB-231 cells by transfection with REST cDNA and observed decreased Matrigel invasion (Fig. S1F). These data confirm a negative correlation between REST expression and invasiveness^2^,^4^.

### An alternatively spliced transcript of REST, REST-003, positively correlates with invasiveness

**Figure 1:**
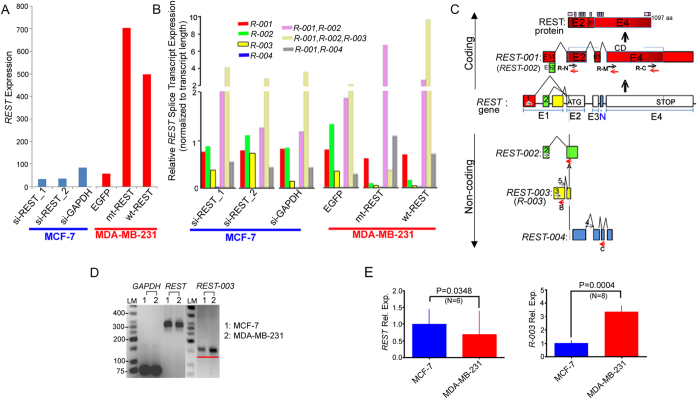
Altering *REST-003* ncRNA expression in si-REST-treated MCF-7 and REST-overexpressed MDA-MB-231 cells. *REST* expression was calculated as the number of reads normalized to the median number of reads for all genes in each sample. (**A**) Expression of *REST* in siRNA-treated MCF-7 and REST-overexpressed MDA-MB-231 cells. (B) Relative expression of each *REST* splice transcript was normalized to total *REST* expression and transcript length in siRNA-treated MCF-7 and REST-overexpressed MDA-MB-231 cells. Some exons in the gene model are shared between multiple transcripts (Fig. S2A). For these, relative expression was calculated as the percentage of *REST-*aligned reads assigned to a specific transcript/transcript-group normalized by the mappable length of the transcript or transcript-group. (**C**) Schematic diagram of the *REST* gene and its splice variant transcripts, including illustrations of annotated *REST* exons and locations of primers employed for the identification of *REST* splice variants. The constitutive transcript (*REST-001*) and alternative spliced variants are shown in red, *REST-002* in green, *REST-003* in yellow, and *REST-004* in blue. *REST-001* and/or *REST-002* can produce the REST protein (wt-REST) that has the complete coding region containing 9 zinc fingers with DNA binding activity (purple) and two repressor domains (pink) necessary for recognizing the RE1 elements and exhibiting repressor function. Forward and reverse primers are indicated by right (numbers) and left (letters) arrows, respectively. (**D) (E**) Detection (**D**) and expression levels (**E**) of ncRNA *REST-003* (3-B primer pair) and coding REST RNA (R-N or R-M primer pair) in MCF-7 and MDA-MB-231 cells by qRT-PCR. (**D**) Following PCR amplification, samples were loaded on 4% agarose gel with a 100-bp marker. (**E**) Expression levels were normalized to GAPDH, CyclophilinA, and/or Actin, converted to MNE, and presented as Relative Expression (Rel. Exp.) after normalization to control samples. Biological replicates are shown on the bar graph as mean plus SEM (paired t-test).

**Figure 2:**
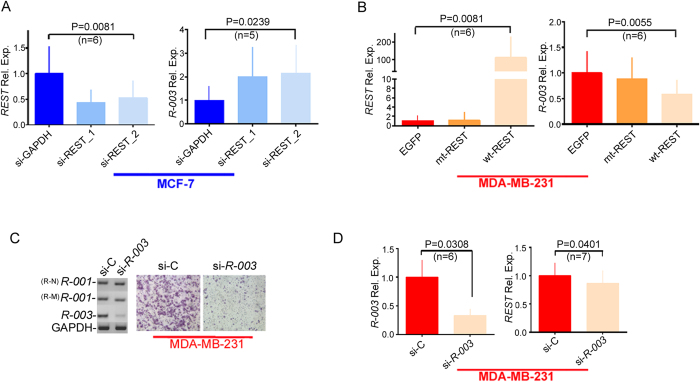
Effect of REST on *REST-003* ncRNA expression in cells, and effect of si-*REST-003* on MDA-MB-231 invasiveness. (**A**) (**B**) Effect of REST on expression of *REST-003* ncRNAs in si-REST-treated MCF-7 (**A**) and REST-overexpressed MDA-MB-231 cells (**B**) by qRT-PCR. MCF-7 cells treated with a non-REST siRNA (si-GAPDH) (A), and MDA-MB-231 cells transfected with EGFP or mt-REST cDNA^6^ (lacking two repressor domains) (B) served as controls. ‘*REST* Rel. Exp.’ refers to *REST‐001* (primer set R‐N). (**C**) Detection of *REST-003* ncRNA by qRT-PCR (left) and invasiveness by a Matrigel invasion chamber (right) after treating MDA-MB-231 cells with si-*REST-003*. (**D**) Reduced *REST-003* expression by si-*REST-003* treatment in MDA-MB-231 cells using qRT-PCR. More than 50% of *REST-003* transcripts were reduced by si-*REST-003* relative to si-C (scramble). REST transcript expression [*REST-001* (*R-001*), R-M primer pair] was not changed by si-*REST-003* (si-*R-003*) treatment. For qRT-PCR data, expression levels were normalized to GAPDH, CyclophilinA and/or Actin, converted to MNE, and presented as Rel. Exp. after normalization to control cells (A, B) or si-C samples (D). Biological replicates are shown on the bar graph as mean plus SEM [one-way ANOVA with Friedman test for multiple comparisons for (A, B) and paired t-test for (D)].

To identify candidate genes that mediate invasiveness, we performed RNA-sequencing (RNA-seq) analysis (GEO accession# GSE63610) of MCF-7 and MDA-MB-231 cells. RNA-seq data suggested downregulation of *REST* transcript levels in MCF-7 cells following treatment with 2 different siRNAs designed to target the protein coding region of REST. *REST* expression also increased when REST cDNA was transfected into MDA-MB-231 cells (Fig. 1A). These changes were confirmed by measuring *REST* expression using qRT-PCR in multiple biological replicates (Fig. 2A,B; Fig. S1D). REST transcript levels did not differ significantly between the two cell lines (Fig. 1D,E).

We estimated the relative expression of each alternatively spliced form of REST based on the EMBL gene model using our RNA-seq pipeline^22^,^23^ (Fig. 1B). We found an alternatively spliced product (ASP) of REST, *REST-003*, whose expression was low in MCF-7 and high in MDA-MB-231 cells (Fig. 1B, Fig. S2A). Due to the complex nature of REST alternative splicing in cancer cells^14^ as well as the small size of RNA-seq reads, we performed additional experiments to confirm this result and determine its role in invasiveness.

Only four REST ASPs are catalogued in the Ensembl Human Genome Browser database (version 75; http://uswest. ensembl.org/ index.html). We confined our analysis to these forms (*REST-001, REST-002, REST-003, and REST-004*), which are illustrated schematically in Fig. 1C along with the *REST* gene (also see Fig. S2B and Supplementary Information). A translation initiation codon is present in Exon 2 (E2). *REST-001* and *REST-002* produce full-length REST protein but contain different 5’ untranslated regions (UTRs). *REST-004* lacks the E2 initiation codon and may thus produce non-coding RNA (ncRNA). *REST-003* contains the initiation codon but lacks other parts of the E2 coding sequence. Since no available data identify *REST-003* as a protein-coding gene, we analyzed the structures of the REST ASPs with qRT-PCR, using specific primers to distinguish the presence or absence of the E2 initiation codon (Fig. 1C). Primers flanking the E2 initiation codon or the middle part of the coding region (R-N and R-M primer pairs) detected high *REST* expression in MCF-7 cells and low expression in MDA-MB-231 cells (Fig. 1D,E). We obtained a similar result with primers flanking the E2 initiation codon but confined to sequences present only in *REST-003* (5-A primer pair; Fig. S3A, B). When testing primers that exclude the initiation codon but are specific to *REST-003* (3-B primer pair), we observed low expression in MCF-7 cells (Fig. 1D,E) and other weakly invasive breast cancer cell lines (Fig. S4A, B), and high expression in MDA-MB-231 cells (Fig. 1D,E, Fig. S4A, B). Similarly, the invasive bladder cancer cell line, T24/83, expressed *REST-003* at higher levels than the non-invasive RT112/84 line (Fig. S4C, D). These results support our RNA-seq data and suggest that only ncRNA derived from *REST-003* correlates positively with invasiveness.

### *REST-003* expression is negatively controlled by REST

We next established the effect of REST modulation on *REST-003* expression using RNA-seq and qRT-PCR. Both methods indicated enhanced *REST-003* expression (>2 fold) following REST downregulation in MCF-7 cells (Fig. 1B, Fig. 2A). Expression of the potential coding RNAs (*REST-002*, primer pair 2-A or REST-003, primer pair 5-A) decreased relative to control expression (Fig. S3A), a pattern similar to that of *REST-001* (Fig. 2A and Fig. S3A). Conversely, overexpression of *REST* in MDA-MB-231 cells resulted in decreased *REST-003* expression (Fig. 1B, Fig. 2B, and Fig. S3B). These results suggest that increased expression of *REST-003* ncRNAs (following loss of REST) may mediate breast cancer cell invasiveness.

### *REST-003* plays an important role in regulating invasiveness

To verify the role of *REST-003* in controlling invasiveness, we knocked down its expression in MDA-MB-231 cells with siRNA (si-*REST-003*). The si-*REST-003*-treated MDA-MB-231 cells exhibited decreased *REST-003* expression (>50%) and reduced Matrigel invasion (>50%) relative to control cells treated with scrambled RNA (si-C; Fig. 2C,D). Treated cells did not show a change in *REST-001* expression (Fig. 2C,D). This implicates a primary role for *REST-003* in regulating invasiveness that is, at least in part, independent of REST protein expression.

### *REST-003* expression is controlled by an uncharacterized serine/arginine repeat-related protein, SRRM3, whose expression may be under REST-mediated transcriptional control

**Figure 3:**
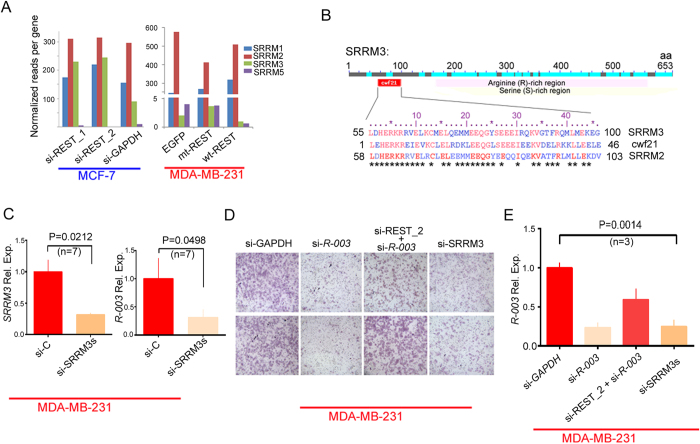
Effect of REST on SRRM3 expression in cells, and effect of SRRM3 on REST-003 ncRNA expression and invasiveness in MDA-MB-231 cells. (**A**) Expression of different SRRM subfamilies in siRNA-treated MCF-7 and REST-overexpressed MDA-MB-231 cells by our pipeline analysis^22^,^23^ of RNA-seq. (**B**) N-terminal sequences of SRRM3, cfw21 (S. cerevisiae), and SRRM2 (H. sapiens) were compared using the ClustalW2 program. Identical residues in cwf21 domains are in red font for all three SR-related proteins. Stars indicate identical residues in SRRM3 and SRRM2. (**C**) Effect of si-SRRM3 on *REST-003* ncRNA expression in MDA-MB-231 cells using qRT-PCR. (**D) (E**) Effect of different siRNA treatments on MDA-MB-231 Matrigel invasiveness (D) and *REST-003* ncRNA expression (E). Expression levels were normalized to GAPDH, CyclophilinA and/or Actin, converted to MNE, and presented as Rel. Exp. after normalization to control cells. Biological replicates are shown on the bar graph as mean plus SEM [paired t-test for (C) and one-way ANOVA with Dunnet test for multiple comparisons for (E)].

We next questioned how REST downregulation could result in increased *REST-003* expression. In neuronal cells, REST transcript is alternatively spliced to produce a REST4 protein, which activates gene expression by competing with REST for RE-1 DNA binding sites^24^. This alternative splicing is mediated by a neural-specific serine/arginine (SR) repetitive matrix 4 protein, SRRM4 (also known as nSR100)^25^. We did not detect any SRRM4 expression by RNA-seq following REST overexpression in MDA-MB-231 cells or in MCF-7 cells treated with si-REST_2 (Fig. 3A). There was, however, a significant change in expression of a related gene, SRRM3 (Fig. 3A), which has no previously documented function. *SRRM3* expression increases >2 fold in MCF-7 cells following treatment with si-REST RNAs, though its expression in MCF-7 is much higher than that in MDA-MB-231, as estimated by our standard pipeline analysis^22^,^23^ (Fig. 3A). We validated this increase using qRT-PCR (Fig. S3C and Table S1). In contrast, *SRRM3* expression decreased by ~3 fold (CuffDiff and our pipeline) in MDA-MB-231 cells following REST overexpression (Fig. 3A). We confirmed these data with qRT-PCR (Fig. S3D).

SRRM3 contains a cwf21 domain, suggesting interaction with SR proteins^26^. It also contains SR-rich domains scattered throughout its sequence (Fig. 3B). However, it lacks a canonical RNA recognition motif (RRM) thought to be necessary for alternative splicing. SRRM3 may thus be an “SR-related protein”^27^ that enhances transcription not by splicing, but in a manner similar to the RSR-2 protein in *C. elegans*^28^. We hypothesized that increased *REST-003* expression and invasiveness were mediated by increased levels of SRRM3 following REST downregulation. To support this notion, when *SRRM3* expression is suppressed in MDA-MB-231 cells using siRNA (si-SRRM3), we observed lower *SRRM3* and *REST-003* expression (Fig. 3C). Importantly, *SRRM3* suppression also reduced MDA-MB-231 Matrigel invasiveness (Fig. 3D). Co-transfection of MDA-MB-231 cells with si-*REST-003* and si-REST_2 eliminated the change in *REST-003* expression as well as the reduction in invasiveness (Fig. 3D,E), suggesting that SRRM3 regulatory control of *REST-003* is positioned downstream of REST protein.

### *REST-003* is processed into mid-size non-coding RNAs as both sense and anti-sense sequences

**Figure 4:**
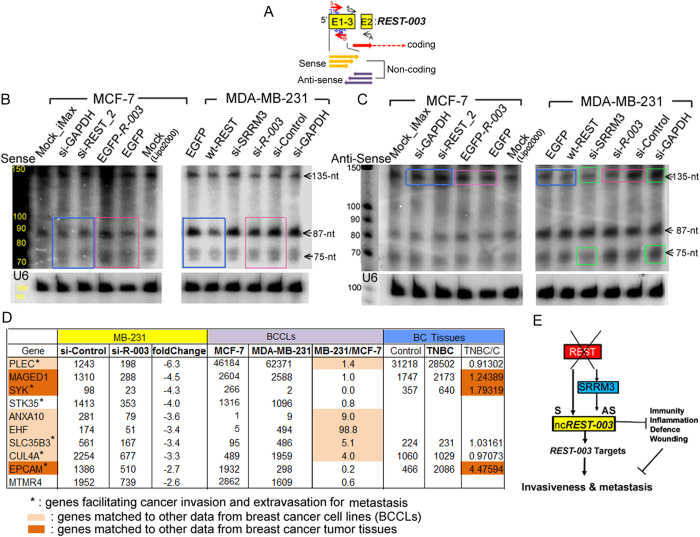
Expression pattern of *REST-003* and its downregulating effect on MDA-MB-231 cells. (**A**) Schematic picture of ncRNAs and coding RNAs transcribed from the E1-3 region. Many new ncRNAs that are enriched at the 5’ boundary of the REST gene (E1-3) are predicted as S (yellow) and AS (purple) sequences. (**B) (C**) Differential expression of *REST-003* in MCF-7 and MDA-MB-231 cells using northern blot analysis. RNA samples were prepared from each cell line transfected with controls and different siRNAs, as indicated. Hybridizations were performed using^32^ P-labeled DNA oligonucleotide probes complementary to the S and AS transcripts probes: AS (B*) sequence of E1-3 for S (B) and S (3*) sequence for AS (C) detection. Human U6 RNA (~105 nt) was probed as an internal control. (**D**) Genes downregulated by *REST-003* downregulation using RNA-seq analysis. Downregulated gene expression in si-*REST-003*-treated cells is shown using DESeq from our pipeline (adjusted P-value < 0.05; yellow). The downregulated genes were compared with published RNA-seq data^35^ from MCF-7 and MDA-MB-231 cell lines (BCCLs, purple), 42 triple negative (TNBC) tissues, and 58 non-malignant control tissues (blue). (**E**) Schematic of the regulatory interactions among REST, *REST-003*, and SRRM3 that may coordinate gene regulation required for development of the invasive phenotype.

*REST-003* appears to be expressed as a ~150-nt-long mid-size non-coding RNA (ncRNA) positioned within the first exon of REST mRNA (Fig. 1B,C). Recent findings reveal many new small- and mid-size ncRNAs that are enriched at the 5’ boundaries of some human genes^15^,^16^. We investigated the potential presence of a cluster of *REST-003* ncRNAs using northern blot analysis of the 5’ region of REST (E1-3 region; Fig. 4A) and found several ncRNAs derived from this region (Fig. S5). Sequences with a length of ~70–150 nt were especially enriched in MDA-MB-231 cells and include both sense (S) and anti-sense (AS) sequences (Fig. 4B,C), an expression pattern similar to that of PASRs that are not yet functionally defined^15^,^16^. The ~75- and ~87-nt-long *REST-003* S and the ~75-, ~87-, and ~135-nt-long *REST-003* AS ncRNA sequences were most highly expressed in MDA-MB-231 relative to MCF-7 cells. Since REST modulation affects *REST-003* expression (Fig. 2A,B), we performed northern analysis in MDA-MB-231cells overexpressing REST and in MCF-7 cells treated with si-REST_2. We found expression of the ~75- and ~87-nt *REST-003* S and the ~135-nt *REST-003* AS ncRNAs to be negatively regulated by REST (Fig. 4B,C, blue boxes).

We also investigated the levels of the ncRNAs following treatment of MDA-MB-231 cells with si-SRRM3. While expression of the ~75- and 135-nt *REST-003* AS ncRNAs were downregulated, *REST-003* S ncRNA was unaffected (Fig. 4B,C, green boxes). Additionally, at least five larger (>200 nt) *REST-003* S and AS bands were highly expressed in MCF-7 cells, similar to *REST-001* (Fig. S5). Notably, we did not detect any ~21–23 nt double-stranded RNAs potentially derived from Dicer processing of *REST-003*. Taken together, our data indicate at least eight RNA variants derived from REST primary transcript (Fig. S5). Three appear to be ncRNAs that likely promote invasiveness (Fig. 4B,C). The other five could potentially code for modified proteins and/or produce other ncRNAs that inhibit invasiveness, similar to REST protein.

### *REST-003* mid-size non-coding RNAs are involved with immune, defense, wounding, and inflammatory responses, as well as cancer cell invasion and/or extravasation for metastasis

We next sought a link between *REST-003* expression, REST target genes, and pathways related to invasion by performing RNA-seq analysis (GEO accession# GSE63610) of MDA-MB-231cells treated with si-*REST-003* (targeting the E1-3 region). Fifty-six genes from DESeq were differentially expressed in the treated versus control samples (Fig. 4D, Fig. S6A). Ten were downregulated in the treated sample, while the other 46 were upregulated. Six of the downregulated genes (PLEC, SYK, STK35, SLC35B2, CUL4a, and EPCAM) are known to facilitate cancer cell invasion and/or extravasation for metastasis^29^,^30^,^31^,^32^,^33^,^34^ (Fig. 4D). We compared these genes with published RNA-seq data from breast cancer cell lines and tissues^35^. Five genes (PLEC, ANXA10, EHF, SLC4A, CUL4A) were expressed highly in MDA-MB-231 relative to MCF-7 cells, and three (MAGED1, SYK, EPCAM) were expressed more in triple-negative tissues relative to non-invasive control tissues (Fig. 4D). We classified the 46 upregulated genes in the treated sample with DAVID functional analysis (Fig. S6A, B) and found more than 20% to be related to immune, defense, wounding, and inflammatory responses (Fig. S6C). Interestingly, neither REST nor its canonical neuronal target genes was affected by knockdown of *REST-003* ncRNAs. These results indicate that *REST-003* ncRNAs play an important role in cancer cell invasion that is largely independent of REST and REST target gene function.

## Discussion

Many classes of ncRNAs associated with the transcriptional start sites (TSSs) of genes have been described^18^. Some examples include PASRs, TSS-associated RNAs (TSSa-RNAs), promoter upstream transcripts (PROMPTs), and transcription initiation RNAs (tiRNAs). Though the biological functions of these classes are poorly defined, most are believed to be involved in transcriptional regulation. In this study, we identified a new class of mid-size ncRNAs derived from a specific alternative splice form of the REST gene (*REST-003*). We propose that these ncRNAs provide a key regulatory function by controlling expression of genes important for cancer cell invasiveness. They may thus represent a new functional example of a PASR-like process (Fig. 4E).

One key technical aspect of our approach was to perform RNA deep sequencing without first selecting polyadenylated RNA. This allowed identification of transcripts derived from the first exon of an alternatively spliced REST transcript (*REST-003*), which are apparently further processed into mid-size (30–200 nt) ncRNAs. As expected from alternative splicing, *REST-*003 levels correlate negatively with those of *REST-001* (the protein coding form of the *REST* gene). More importantly, we found that *REST-003* levels correlate positively with cancer cell invasiveness in several cancer cell lines.

Subtypes of breast cancer are often classified with markers such as estrogen receptor (ER), progesterone receptor (PR), and HER2 that relate to clinical outcomes. At least four subtypes are recognized based on different expression patterns for these markers^36^,^37^,^38^. Ranked from most invasive to least invasive, these include^36^,^39^: basal-like triple negative (ER^−^, PR^−^, HER2^−^) = HER2^+^ > luminal B (ER^+^, PR^+/−^, HER2^+^) > luminal A (ER^+^, PR^+/−^, HER2^−^). We found that this order correlates well with levels of *REST-003* (Fig. S4A, B). Low *REST-003* levels were observed in MCF-7, T47D (luminal A), BT474 (luminal B), SKBR3 (HER2^+^) and MDA^-^MB-468 (basal-like) cell lines, all of which are non-invasive in a Matrigel assay (Fig. S4A, B). Conversely, high *REST-003* levels were observed in basal-like triple negative and invasive MDA-MB-231 cells (Fig. 1D,E; Fig. S4A, B). Similarly, an invasive bladder cancer cell line (T24/83) exhibited high levels of *REST-003* relative to non-invasive RT112/84 bladder cancer cells (Fig. S4C, D). These results suggest that *REST-003* could be used as a prognostic marker to access invasive potential of breast and other types of cancer cells. Further studies will be required to establish this more firmly.

Our data suggest a possible mechanism for regulating alternative splicing of the REST primary transcript into *REST-001* (or alternative protein coding forms) and *REST-003*. We found that REST itself negatively regulates expression of SRRM3, which suggests a positive association between the expression of SRRM3 and *REST‐003* (Fig. 3A,C). SRRM3 has not yet been functionally characterized. We detected increased SRRM3 expression following downregulation of REST in MCF-7 cells and decreased expression following overexpression of REST in MDA-MB-231 cells (Fig. 3A), suggesting direct or indirect control of REST alternative splicing in addition to autoregulation via REST.

The alternative splicing networks in neuronal cells and cancer cells are clearly different. Neuronal cells express SRRM4, a known splicing regulator of REST^25^, while breast cancer cells lack SRRM4 expression (Fig. 3A) (with the exception of small cell lung cancer^5^). As a consequence, splicing in invasive MDA-MB-231 cells is not regulated by SRRM4. We propose that *REST-003* expression is rather controlled by SRRM3. Regulatory networks for alternative splicing are complex and are still not fully defined^40^. Additional work is required to directly demonstrate this proposed mechanism favoring invasiveness and *REST-003* in cancer cells.

Our findings show that REST downregulation results in increased production of *REST-003*, which is processed into many different sizes of ncRNAs (Fig. 4B,C). At least 3 of these processed ncRNAs are more prevalent (75- and 85-nt S, and 135-nt AS) (Fig. 4B,C). Conversely, overexpression of REST results in a decrease in these same ncRNA fragments (Fig. 4B,C). These data suggest that further characterization focusing on these specific *REST-003*-derived ncRNAs may be the most significant factor controlling invasiveness. Interestingly, only reduction of the 135-nt AS product is observed following downregulation of SRRM3 (Fig. 4C, green boxes). Perhaps other splicing factors are involved in regulation, or other processing pathways remain to be discovered (Fig. 4E). Further studies could establish whether they act together or separately in regulating invasiveness as well as whether they are important in other cancer types.

Following si-*REST-003* treatment of MDA-MB-231 cells, an impressive subset of invasive-related genes was downregulated (PLEC, SYK, STK35, SLC35B2, CUL4A, and EPCAM). These genes are known to be important not only for cell line invasiveness (Fig. 4D), but also for cancer tumor progression to metastasis^29^,^30^,^31^,^32^,^33^,^34^. This suggests that *REST-003* ultimately plays an important role in controlling invasiveness through a gene expression program. Other RNA-seq data from breast cancer cell lines and tissues^35^ provide supporting information for this hypothesis. For example, PLEC, ANXA10, EHF, SLC4A, and CUL4A are expressed at high levels in MDA-MB-231 relative to MCF-7 cells, and MAGED1, SYK, and EPCAM are expressed at higher levels in triple-negative cancer tissues relative to non-invasive control tissues (Fig. 4D). In addition, *REST-003* appears to be involved not only in regulating invasiveness, but also in immune, defense, wounding, and inflammatory responses by upregulating expression of genes in these pathways (Fig. S6). Each of these genes may thus be regulated in different ways (i.e., increased or decreased) by the *REST-003* mid-size ncRNAs. Our RNA-seq data place the focus on *REST-003*-derived mid-size ncRNAs.

In conclusion, a mechanistic role for *REST-003* and SRRM3 in regulating cancer cell invasiveness suggests novel therapeutic approaches for limiting breast (and other types of) cancer invasion and metastasis.

## Methods

### Cell culture, transfection, qRT-PCR and northern blot

MDA-MB-231 and MCF-7 cancer cell lines were obtained and cultured as described previously^41^. Lipofectamine 2000 (Invitrogen) was used for all transfection experiments unless otherwise specified. For siRNA transfection, we used DharmaFect (Thermo Scientific) or RNAiMax (Invitrogen) according to the manufacturer’s instructions. siRNA sequences are provided in Supplementary Table 1.

For qRT-PCR, cDNAs were made from the total RNAs treated with DNase as described in our previous study^42^. Gene-specific q-PCR primer or probe sets (Table S1) for human genes, GAPDH, CyclophilinA, and Actin, and equivalent amounts of cDNA generated as a template were used for qRT-PCR. Reactions were performed for each sample using SSoFast EvaGreen Supermix (Bio-Rad) or TaqMan Universal PCR Master Mix (Invitrogen) with a CFX-96 system (Bio-Rad). For each sample, expression of marker genes was normalized to GAPDH, CyclophilinA or Actin [mean normalized expression (MNE)]. MNEs were normalized to control samples to present relative expression (Rel. Exp.). Northern blots were performed as described previously^43^.

### Matrigel invasion assay

Invasion was measured using BD BioCoat Matrigel Invasion Chambers (BD Biosciences) according to the manufacturer’s instructions, as described in our previous study^41^.

### Library preparation, sequencing, and analysis of RNA-seq

Ribosomal RNA (rRNA) was depleted from 100 ng of total RNA using a Ribo-Zero Magnetic Gold kit (Epicentre) according to the manufacturer’s protocol. Libraries from rRNA-depleted samples were prepared using a TruSeq RNA Sample Preparation kit v2 (Illumina) following the recommended protocol starting from the RNA fragmentation stage. Purification of polyadenylated RNA was omitted. Libraries were pooled (4 samples per pool), clustered on cBOT (Illumina), and sequenced on HiSeq2000, each pool in one lane. We performed single-end 100 bp reads. Reads were mapped using TopHat followed by data analysis using Cufflinks and Cuffdiff software, as well as our own pipeline^22^,^23^.

For tissue data, expression reads of downregulated genes by si-*REST-003* treatment were averaged from TNBC and controls by our pipeline^22^,^23^ assay of RNA-seq data^35^ (Table S2) and compared to each other.

### Statistical analysis

Bar graphs show the mean ± SEM of biological replicates (n). Detailed statistical methods are indicated in the figure legends.

## Additional Information

**How to cite this article**: Lee, N.S. *et al.* Non-coding RNAs derived from an alternatively spliced REST transcript (*REST-003*) regulate breast cancer invasiveness. *Sci. Rep.* **5**, 11207; doi: 10.1038/srep11207 (2015).

## Supplementary Material

Supplementary Information
