# Supplementary material for: Non-coding RNAs derived from an alternatively spliced REST transcript (REST-003) regulate breast cancer invasiveness
Source: Sci Rep. 2015 Jun 8;5:11207. doi: 10.1038/srep11207 (PMC4459148; doi:10.1038/srep11207)
Supplement: Supplementary Information [file srep11207-s1.pdf]

Non-coding RNAs derived from an alternatively spliced REST transcript *(REST-003)* regulate breast cancer invasiveness

Nan Sook Lee[1,2*], Oleg V. Evgrafov[3], Tade Souaiaia[3], Adrineh Bonyad[1], Jennifer Herstein[3], Joo Yeun Lee[4], Jihong Kim[3], Yan Ning[5], Marcos Sixto[6], Andrew C. Weitz[7], Heinz-Josef Lenz[5], Kai Wang[3], James A. Knowles[3], Michael F. Press[5], Paul M. Salvaterra[8], K. Kirk Shung[2], Robert H. Chow[1]

SUPPLEMENTARY FIGURE LEGENDS

Fig. S1. Differences in REST expression and invasiveness between MCF-7 and MDA-MB-231cells. (**A**) Invasive potential of each cell line determined by a Matrigel Invasion Chamber assay. MCF-7 cells are normally not invasive, while MDA-MB-231 cells are highly invasive. Purple cells in the images have invaded and moved across the Matrigel barrier. (**B, C**) Expression of *REST* transcript (**B**) and REST protein (**C**) by qRT-PCR and western blot, respectively, in MCF-7 and MDA-MB-231cells. (**D**) Alteration of *REST* mRNA expression by siRNA and cDNA treatment in both cell lines using q-RT-PCR. Approximately 60-80% of REST was reduced by siRNAs against REST (si-REST_1 or si-REST_2) in MCF-7 cells. The transfected MDA-MB-231 cells with wild-type (wt) REST cDNA show higher REST mRNA expression relative to control cells transfected with EGFP cDNA. The transfected MDA-MB-231 cells with a mutant (mt)-REST cDNA lacking the 2 repressor domains unexpectedly exhibit more REST expression relative to wt-REST cDNA-transfected cells. REST-N (R-N) and REST-C (R-C) primers can distinguish expression of wt- and mt-REST expression in MDA-MB-231

cells compared to REST-M (R-M) primers. Their expression levels were normalized to the housekeeping genes, GAPDH and/or Cyclophilin A (''MNE''). Error bars indicate SEM (n = 5 for each experiment). Since si-REST_2 was more effective than si-REST_1, we chose to use si-REST_2 in further experiments. (**E**) Effect of REST downregulation on Matrigel invasiveness in MCF-7 cells. These cells showed invasiveness after si-REST_2 treatment. (**F**) Overexpression of wt-REST reduced invasiveness of MDA-MB-231 cells. Overexpression of mt-REST reduced invasiveness relative to a control (EGFP) but not to the same degree as wt-REST. Representative images are shown in all cases.

Fig. S2. Bioinformatics at the *REST* gene locus. (**A**) Data were retrieved from the UCSC or Ensemble Genome Browser 75 (http://uswest.ensembl.org/ index.html). Annotated *REST* exons and their splicing images are illustrated. The first exon (E1) contains three different parts (E1-1, E1-2 and E1-3) to be spliced out and connected to E2. The constitutive transcript (*REST-001*; E1-1, E2, E3, and E4) and alternative spliced variants are shown in red and different colors, respectively (green for *REST-002*, yellow for *REST-003,* and blue for *REST-004*): *REST-002*; E1-2 to E2, *REST-003*; E1-3 to E2. *REST-004* contains truncated E2, E3, Exon N and truncated E4. (**B**) EnsEMBL_Web_Component_Gene_SpliceImage-Homo_sapiens-Gene-Splice-73-ENSG00000084093.

Fig. S3. Effect of REST modulation on expression of ncRNAs and SRRM3 by qRT-PCR. (**A, B**) Effect of REST downregulation in MCF-7 (**A**) and REST overexpression in MDA-MB-231 (**B**) on expression of ncRNAs by qRT-PCR. Expression of ncRNAs increases following REST downregulation in MCF-7 (**A**) and decreases following REST overexpression in MDA-MB-231 (**B**) relative to the controls. In contrast, expression of coding RNAs produces opposite behavior.

(**C**) Effect of REST downregulation in MCF-7 (left) and REST overexpression in MDA-MB-231 (right) on expression of SRRM3 by qRT-PCR. Expression levels were normalized to the housekeeping genes, GAPDH and/or Cyclophilin A (‘‘MNE’’). Error bars indicate SEM ($n = 2$ or 3 for each experiment). $*p < 0.05$.

Fig. S4. Positive correlation between *REST-003* expression and invasiveness in several breast and bladder cancer cell lines. (**A**) Invasive potential of each cell line was determined by a Matrigel Invasion Chamber assay. Purple cells in the images have invaded and moved across the Matrigel barrier. Cell lines were classified into four subtypes: luminal A, luminal B, HER2$^+$ and basal-like (triple negative). Immunoprofiles of each subtype are provided. (**B**) Expression of *REST-003* in each cell line as determined via q-RT-PCR. *REST-003* was highly expressed in invasive MDA-MB-231 cells but not in other cell lines that exhibit no Matrigel invasion. (**C, D**) Positive correlation of invasiveness to *REST-003* expression in bladder cancer cell lines. T24/83 cells are shown to be invasive by Matrigel Invasion Chamber assays, while RT112/84 are not invasive (**C**). T24/83 cells expressed *REST-003* at higher levels than non-invasive RT112/84 (**D**).

Fig. S5. Northern gel picture of differential expression of *REST-003* in MCF-7 and MDA-MB-231 cells. At least five larger (>200 nt) *REST-003* S and AS bands are highly expressed in MCF-7 cells, similar to *REST-001*.

Fig. S6. Upregulated genes and their pathways following si-*REST-003* treatment in MDA-MB-231 cells. (**A, B**) The level of gene expression in si-*REST-003*-treated MDA-MB-231 cells was

compared with that of control (si-C) cells. Upregulated gene expression in si-*REST-003*-treated cells is shown using DESeq from our pipeline (adjusted *P*-value < 0.05) and functional analysis from DAVID (FDR < 0.05). Different functions of genes are presented as different colors (**A**) and terms (**B**). (**C**) Top pathways upregulated by downregulation of *REST-003* in MDA-MB-231 cell lines are shown using DESeq from our pipeline (adjusted *P*-value < 0.05).

Fig. S1.

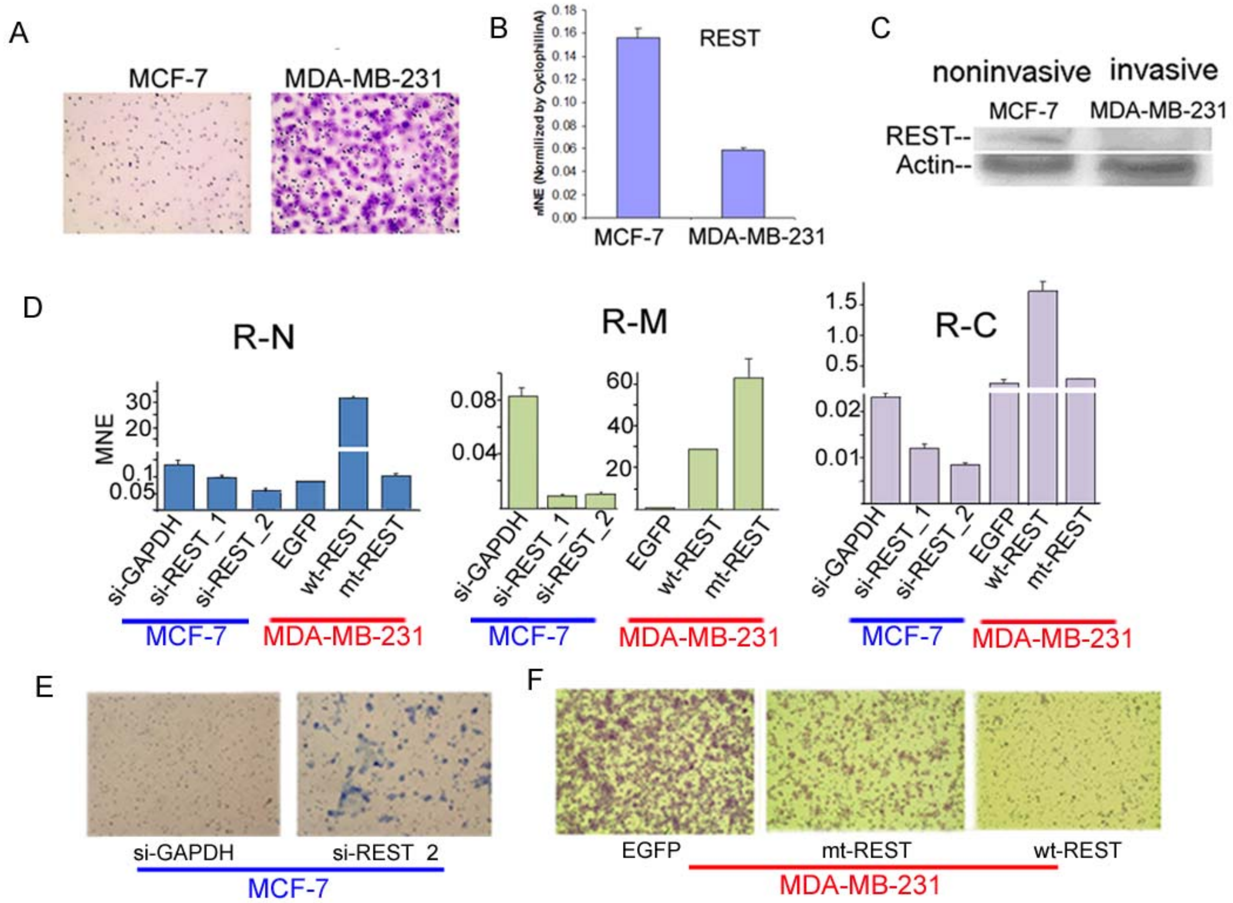

A
MCF-7
MDA-MB-231
B
REST
mNE (Normilized by CyclophilinA)
MCF-7
MDA-MB-231
C
noninvasive
invasive
MCF-7
MDA-MB-231
REST--
Actin--
D
R-N
MNE
si-GAPDH
si-REST_1
si-REST_2
EGFP
wt-REST
mt-REST
MCF-7
MDA-MB-231
R-M
R-C
E
si-GAPDH
si-REST 2
MCF-7
F
EGFP
mt-REST
wt-REST
MDA-MB-231

Fig. S2.

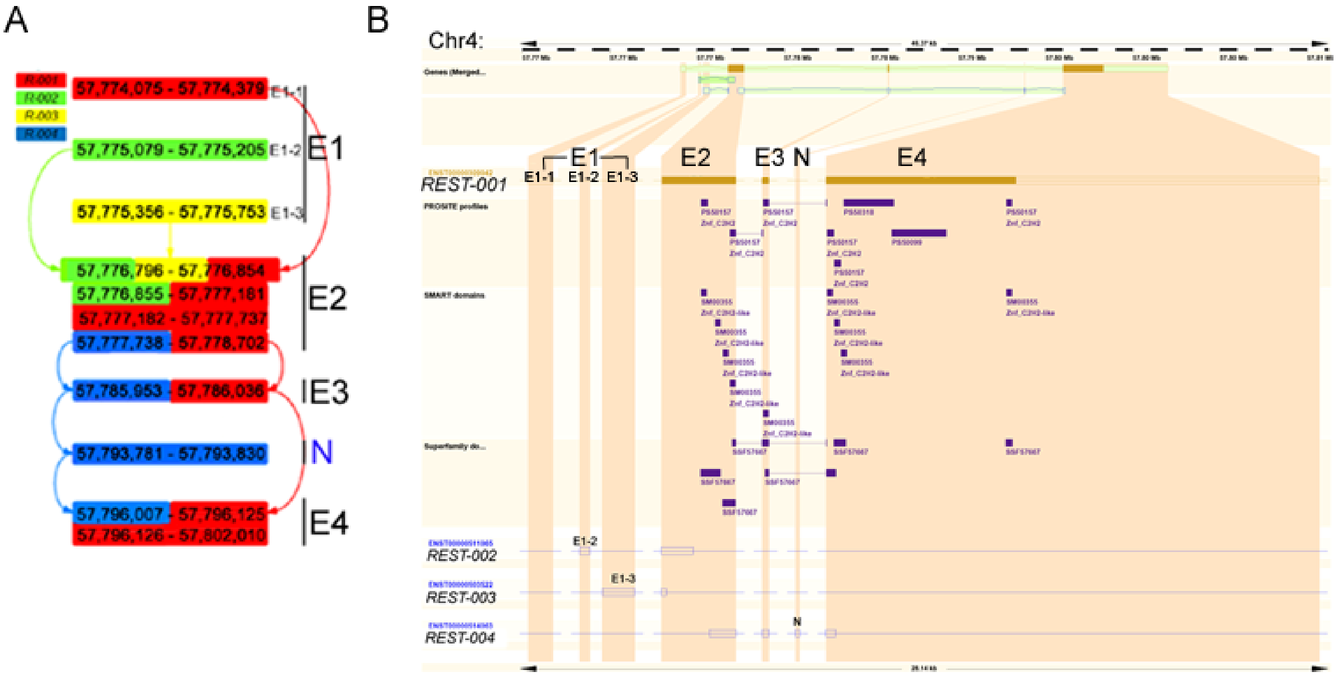
A
R-001
R-002
R-003
R-004
57,774,075 - 57,774,379
E1-1
57,775,079 - 57,775,205
E1-2
E1
57,775,356 - 57,775,753
E1-3
57,776,796 - 57,776,854
57,776,855 - 57,777,181
57,777,182 - 57,777,737
E2
57,777,738 - 57,778,702
57,785,953 - 57,786,036
E3
57,793,781 - 57,793,830
N
57,796,007 - 57,796,125
57,796,126 - 57,802,010
E4
B
Chr4:
E1
E1-1
E1-2
E1-3
E2
E3
N
E4
REST-001
REST-002
E1-2
REST-003
E1-3
REST-004
N

Fig. S3.

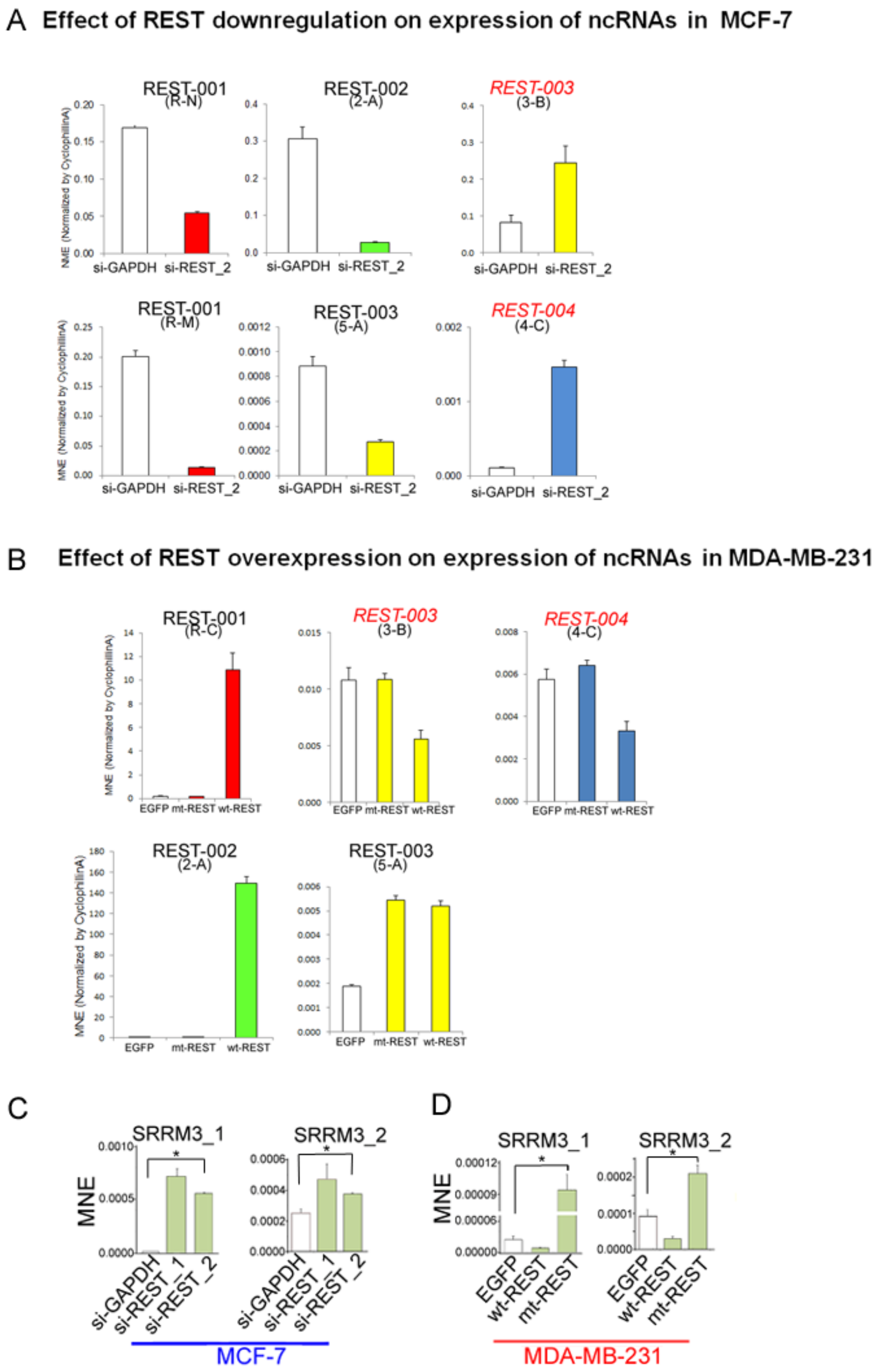

A Effect of REST downregulation on expression of ncRNAs in MCF-7
REST-001 (R-N)
REST-002 (2-A)
REST-003 (3-B)
REST-001 (R-M)
REST-003 (5-A)
REST-004 (4-C)
MNE (Normalized by CyclophilinA)
si-GAPDH
si-REST_2
B Effect of REST overexpression on expression of ncRNAs in MDA-MB-231
REST-001 (R-C)
REST-003 (3-B)
REST-004 (4-C)
REST-002 (2-A)
REST-003 (5-A)
EGFP
mt-REST
wt-REST
C
SRRM3_1
SRRM3_2
MNE
si-GAPDH
si-REST_1
si-REST_2
MCF-7
D
SRRM3_1
SRRM3_2
EGFP
wt-REST
mt-REST
MDA-MB-231

Fig. S4.

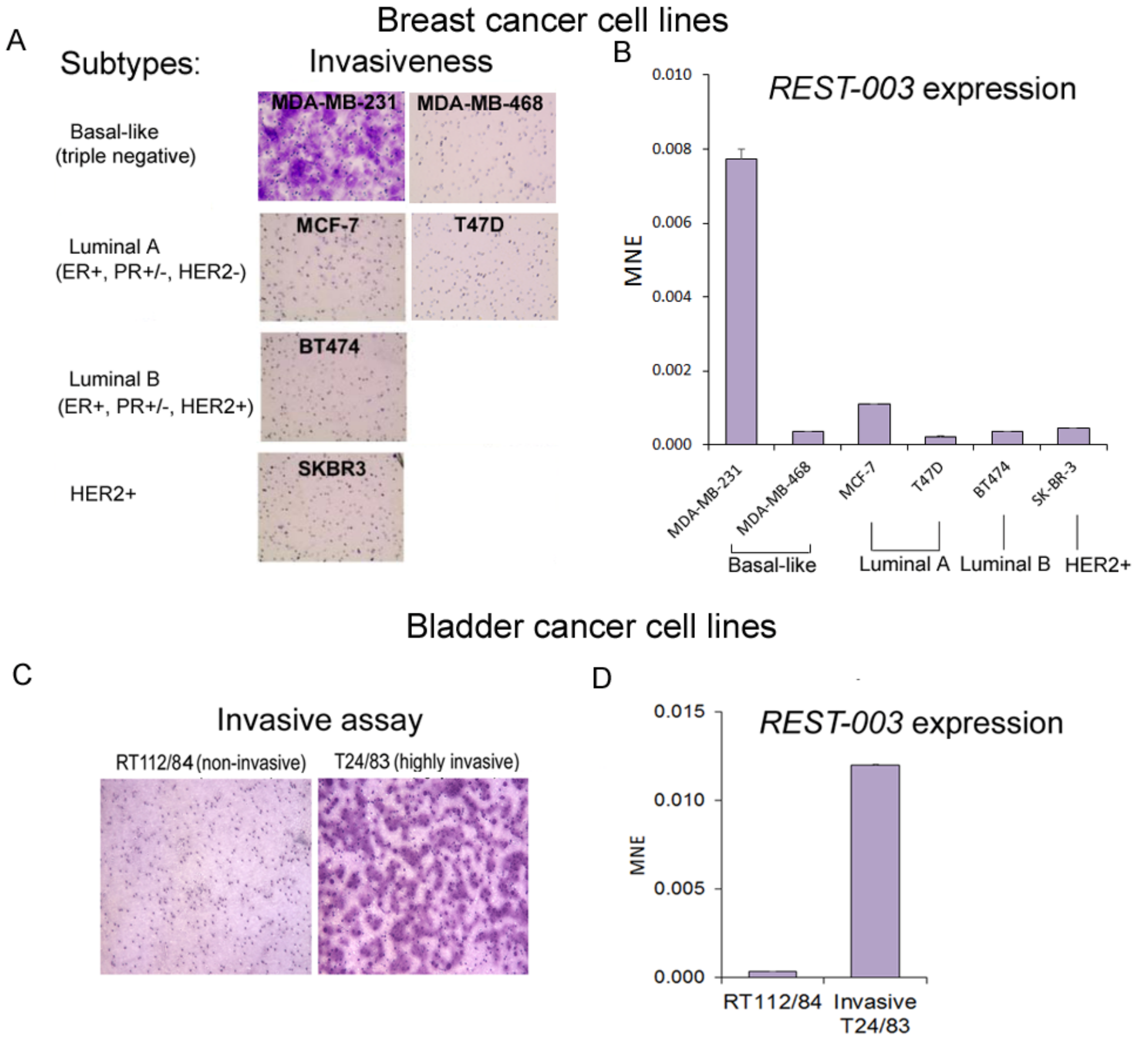

Breast cancer cell lines
A
Subtypes:
Invasiveness
Basal-like
(triple negative)
MDA-MB-231
MDA-MB-468
Luminal A
(ER+, PR+/-, HER2-)
MCF-7
T47D
Luminal B
(ER+, PR+/-, HER2+)
BT474
HER2+
SKBR3
B
REST-003 expression
MNE
0.010
0.008
0.006
0.004
0.002
0.000
MDA-MB-231
MDA-MB-468
MCF-7
T47D
BT474
SK-BR-3
Basal-like
Luminal A
Luminal B
HER2+
Bladder cancer cell lines
C
Invasive assay
RT112/84 (non-invasive)
T24/83 (highly invasive)
D
REST-003 expression
MNE
0.015
0.010
0.005
0.000
RT112/84
Invasive
T24/83

Fig. S5.

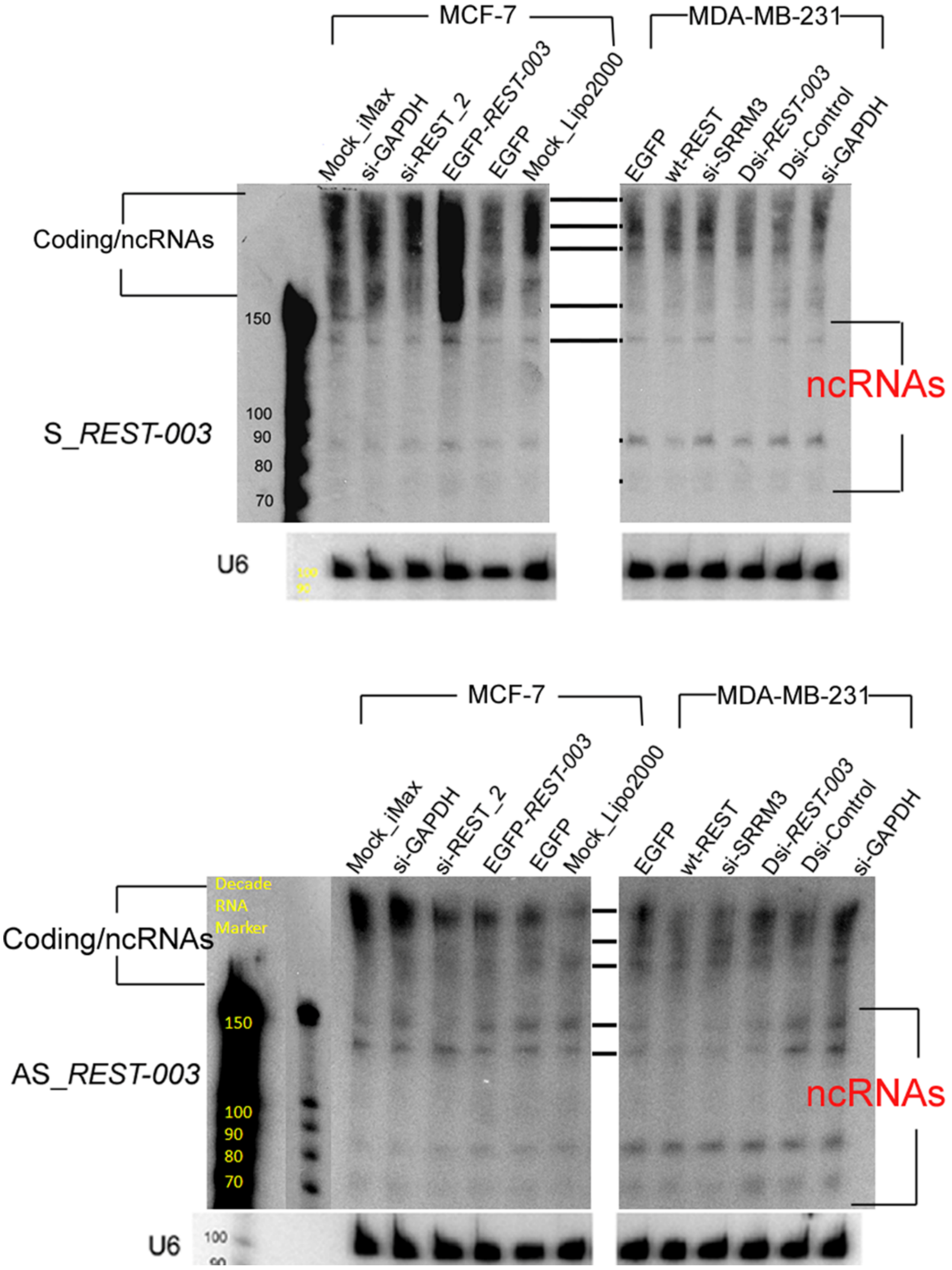

MCF-7
MDA-MB-231
Mock_iMax
si-GAPDH
si-REST_2
EGFP-REST-003
EGFP
Mock_Lipo2000
EGFP
wt-REST
si-SRRM3
Dsi-REST-003
Dsi-Control
si-GAPDH
Coding/ncRNAs
150
100
90
80
70
S_REST-003
ncRNAs
U6
MCF-7
MDA-MB-231
Mock_iMax
si-GAPDH
si-REST_2
EGFP-REST-003
EGFP
Mock_Lipo2000
EGFP
wt-REST
si-SRRM3
Dsi-REST-003
Dsi-Control
si-GAPDH
Decade
RNA
Marker
Coding/ncRNAs
150
100
90
80
70
AS_REST-003
ncRNAs
U6
100

Fig. S6.

A

Gene ID	foldChange	si-C	si-*REST-003*	FA
IFNL1	103.03	0.32	32.81	
CXCL10	21.16	109.15	2309.56	T CR IR InR TLI CD C DR RV CT I InI CA LB
IFNB1	19.41	21.76	422.32	RV CR DR PRI TLI CD CA C
CXCL11	16.34	192.55	3145.36	T CR RW DR IR TLI InR CT CA InR C LB
CCR1	15.75	10.42	164.13	T CR RW DR IR InR CT LB
GBP5	13.50	148.94	2010.69	IR GB GBI GBP-C
APOL3	13.05	19.80	258.30	PRS RN RPK PRC RW DR InR PRI PRPKC
GBP4	11.97	50.75	607.40	IR GB GBI GBP-C
C1S	11.59	38.05	441.20	RW DR IR PRR InR
CASP1	10.25	10.65	109.14	PRS RN RPK PRC PRI CD PRPKC
XAF1	9.76	215.43	2102.98	
CCL5	8.85	12.38	109.53	RV T CR IR PRI IR TLI CC CD C LC DR RV CT I IR CA LB
IDO1	8.81	199.34	1756.14	RW DR PRR InR
IRG1	8.45	21.21	179.14	
GBP1	8.09	680.20	5504.33	IR GB GBI GBP-C
TNFSF10	7.70	39.38	303.15	PRS RN RPK PRC CR IR PRI CA C PRPKC
CD274	7.70	99.18	761.83	IR
RTP4	6.94	24.54	170.17	
IFIT2	6.68	3638.30	24310.01	
TFPI2*	6.42	902.23	5794.15	RW
APOL1	6.38	17.94	114.48	DR IR
GBP1P1	6.31	37.87	238.85	
BST2	6.19	66.50	411.40	RV PRS RN RPK PRC IR PRI PRPKC
IFIT3	6.08	3797.42	23099.19	
TGFBI*	5.99	194.79	1167.37	
TRIM22	5.81	337.29	1959.65	RV IR
PSAT1	5.64	208.05	1173.04	
RSAD2	5.45	3235.33	17632.63	RV DR IR
CEACAM1	5.22	59.99	312.95	
GBP2	5.18	220.93	1144.21	IR GB GBI GBP-C
TMEM171	5.15	21.35	109.93	
IL8	4.75	6784.59	32219.23	T CR IR PRR InR TLI CC C LC DR RW CT I InI CA LB
TLR3	4.65	275.63	1281.89	RV RN RPK PRC IR PRR InR TLI PR PRS DR RW PR InR
CBX1	4.58	649.41	2975.84	
OASL	4.46	1460.41	6518.65	IR
SERPINE1	4.37	265.10	1159.55	RW
MMP13	4.21	342.74	1444.32	
IL1B	4.20	399.83	1677.34	T RN RPK PRC CR IR PRR InR TL CC CD C PR PR LC DR RW CT PR I CA InR LB
HERC5	4.16	1075.89	4478.68	
FNDC3A	3.90	2147.16	8364.72	
CMPK2	3.82	2175.57	8313.51	
ARL6IP1	3.69	951.85	3512.16	
PGAM1	3.61	2165.75	7812.82	
TAP1	3.36	1069.07	3593.28	DR IR
PMAIP1	2.81	972.26	2736.43	
IL6	2.81	192.71	540.86	RV T RPK PRC CR IR PRR InR TL CC CD C PR PR LC DR RW CT CA LB

B

Symbol	Term
IR	GO:0006955~immune response
DR	GO:0006952~defense response
RW	GO:0009611~response to wounding
InR	GO:0006954~inflammatory response
PRRS	GO:0048584~positive regulation of response to stimulus
RPKC	GO:0010627~regulation of protein kinase cascade
PRST	GO:0009967~positive regulation of signal transduction
CR	hsa04060:Cytokine-cytokine receptor interaction
PRCC	GO:0010647~positive regulation of cell communication
TLR	hsa04620:Toll-like receptor signaling pathway
CA	GO:0005125~cytokine activity
T	GO:0042330~taxis
CT	GO:0006935~chemotaxis
PRPKC	GO:0010740~positive regulation of protein kinase cascade
C	cytokine
RNF	GO:0043122~regulation of I-kappaB kinase/NF-kappaB cascad
RV	GO:0009615~response to virus
PRNF	GO:0043123~positive regulation of I-kappaB kinase/NF-kappaB
I	inflammatory response
CDSP	hsa04623:Cytosolic DNA-sensing pathway
LC	GO:0030595~leukocyte chemotaxis
CC	GO:0060326~cell chemotaxis
GBP-C	IPR003191:Guanylate-binding protein, C-terminal
GBP1	PIRSF005552:guanine nucleotide-binding protein 1
GBP-N	IPR015894:Guanylate-binding protein, N-terminal

C

DESeq: Top pathways upregulated by downregulation of *REST-003*

Category	Term	Count	%
GOTERM_BP_FAT	GO:0006955~immune response	21	39
GOTERM_BP_FAT	GO:0006952~defense response	15	28
GOTERM_BP_FAT	GO:0009611~response to wounding	13	24
GOTERM_BP_FAT	GO:0006954~inflammatory response	11	20
GOTERM_BP_FAT	GO:0048584~positive regulation of response to stimulus	9	17
GOTERM_BP_FAT	GO:0010627~regulation of protein kinase cascade	9	17
GOTERM_BP_FAT	GO:0009967~positive regulation of signal transduction	9	17
KEGG_PATHWAY	hsa04060:Cytokine-cytokine receptor interaction	9	17
GOTERM_BP_FAT	GO:0010647~positive regulation of cell communication	9	17
KEGG_PATHWAY	hsa04620:Toll-like receptor signaling pathway	8	15
GOTERM_MF_FAT	GO:0005125~cytokine activity	8	15
GOTERM_BP_FAT	GO:0042330~taxis	8	15
GOTERM_BP_FAT	GO:0006935~chemotaxis	8	15
GOTERM_BP_FAT	GO:0010740~positive regulation of protein kinase cascade	8	15
SP_PIR_KEYWORDS	cytokine	8	15
GOTERM_BP_FAT	GO:0043122~regulation of I-kappaB kinase/NF-kappaB cascade	7	13
GOTERM_BP_FAT	GO:0009615~response to virus	7	13
GOTERM_BP_FAT	GO:0043123~positive regulation of I-kappaB kinase/NF-kappaB cascade	7	13
SP_PIR_KEYWORDS	inflammatory response	6	11
KEGG_PATHWAY	hsa04623:Cytosolic DNA-sensing pathway	6	11
GOTERM_BP_FAT	GO:0030595~leukocyte chemotaxis	5	9
GOTERM_BP_FAT	GO:0060326~cell chemotaxis	5	9
INTERPRO	IPR003191:Guanylate-binding protein, C-terminal	4	7
PIR_SUPERFAMILY	PIRSF005552:guanine nucleotide-binding protein 1	4	7
INTERPRO	IPR015894:Guanylate-binding protein, N-terminal	4	7

Table S1. Primers and si-RNAs used in this study. Details are in the Supplementary Information.

ASP REST and SRRM3 transcripts	Description	F/R	Sequences
REST	REST-N (R-N)	Forward	5'-CTTCTGGAGGAGGAGGGCTGTTTAC
		Reverse	5'-CATAATAAGCTGAGGTGCGGCCAG
	REST-M (R-M)	Forward	5'-GAACTCATACAGGAGAACGCC
		Reverse	5'-GAACTGCCGTGGGTTCACA
	REST-C (R-C)	Forward	5'-TGAAGAACCAGTTTCACCAATGCTTC
		Reverse	5'-GCTACAATGGCAGCAAATGAGTCTCAG
REST-001	#1 (E1-1)	Forward	5'-AGAAAAGTAGTCGGAGAAGGAGCGG
	#9 (E1-1 / E2)	Forward	5'- GAGGAAGGCCG / AATACAGTT**ATG**G
REST-002	#2 (E1-2 / E2)	Forward	5'- GACGCCGGCTGCGCG AATACAGTTATGGC
REST-003	#3 (E1-3)	Forward	5'-AGTGTCGGGGCGACTCCCG
	#5 (E1-3)	Forward	5'-GTCGATGTTGGGCCAAATTACCCAATAGC
	#3* (short)	Forward	5'-GTAAATGTGTGCAGTGAGCGGGC
AS REST-003	(same sequences as Sen	Forward	5'-ATACCAAACACAAAGCAGCTCTTTG
		Reverse	5'-GGCGACTCCCGCGAGTTGGTGTG
REST-004	#4 (E2)	Forward	5'-CACACCAGAGCTGGGGATAATGAGC
	#6 (*E2 / E3)	Forward	5'- AGAACTCATACAG GAGAACGCCCATATAAATG
	R4	Forward	5'-CATTCAGTGGGGTATGGATACC
REST-001	A	Reverse	5'-GTAAACAGCCCTCCTCCTCCAGAAG
REST-003	B	Reverse	5'-GGCATTCCTAACTGAAATAGG
	B* (short)	Reverse	5'-CATTCGGCCATTTTCTCAAAATAC
REST-004	C (=R4)	Reverse	5'-GCTTCTCACCCATCTAGATCAC
SRRM3_1		Forward	5'-TGGTGAAGCGCGCGCACCGCGAGATCC
		Reverse	5'-GAATGTCCCCACTTTCTGCCGAATC
		Reverse-1	5'-ATCTCCTCCTCCGAATACCCCTGCTC
SRRM3_2		Forward	5'-TCCTGGAGCTCCAGCCGCTCGCCC
		Reverse	5'-CTCAGAGTGCCTTGCGCGGCCCTCG

**Transcripts**	**si-RNA name**		**Target Sequences**
REST	si-REST_1 (Dharmacon)	#1 (E4)	GGUGAAACUUUAAAUGGUA
	(ON-TARGETplus SMARTpool L-006466-00)	#2 (E4)	GAAUCUCACUGGUAUAAAU
		#3 (E4)	CAUCCUACUUGUCCUAAUA
		#4 (E3)	AGACAUAUGCGUACUCAUU
	si-REST_2 (Dharmacon)	#1 (E2)	CGACAUGUAUGACUUGCAU
	(siGENOME SMARTpool M-006466-02)	#2 (E4)	GGGCCUAAACCUCUUAAUU
		#3 (E4)	GAUGGAGGGUGCCCAGAUA
		#4 (E4)	CAGUAUAGUUUGUGAAAUG
REST-003	*Dsi-REST-003 (IDT)	E1-3	GCAAAGAGCUGCUUUGUGUUUGGUA
	*Dsi-C (Scramble) (IDT)		CGUUAAUCGCGUAUAAUACGCGUAU
	*AS Dsi-REST-003 (same as S)		UUUGCAAAGAGCUGCUUUGUGUUUGGU
SRRM3	si-SRRM3 (Ambion)		CAAAGAGCCGUUACGAACAtt
	*Dsi-SRRM3_1 (IDT)		GGAAGAGACGGCACAGAUCUCGAAG
	*Dsi-SRRM3_2 (IDT)		GCAAGCGUCCUAUUCCAUACUACCG
	si-SRRM3 (Dharmacon)	#1	ACAAAGAGCCGUUACGAAC
	(siGENOME SMARTpool M-016790-01)	#2	GGAGAAGCCAGAUGUGCUG
		#3	CAAAGAGGUCUCAGGGCCA
		#4	AGACUUUGAGGGUGGGCAU
	si-SRRM3_1 (Dharmacon)		AGAAGAAGAGUGUGAAGAAUU
	si-SRRM3_2 (Dharmacon)		GCAUGGAGCUGCAGGAGAUUU

*DsiRNA (Dicer-induced siRNA)

Table S2. Averaged reads of each down-regulated gene expression by si-*REST-003* treatment from 42 TNBC and 58 controls by our pipeline (*1, 2*) assay of published RNA-seq data (*3*).

geneName	CONTROL**	TNBC**	ER^+BC**	UBT_TNBC**	UBT_ER^+**
PLEC	31217.60	28502.40	26492.60	23095.80	27743.00
MAGED1	1747.20	2173.33	2199.40	2030.86	1941.87
SYK	356.80	639.81	359.64	441.95	490.43
STK35 *					
ANXA10*					
EHF*					
SLC35B3	223.60	230.67	157.02	245.38	201.83
CUL4A	1060.20	1029.17	968.24	1154.86	1095.93
EPCAM	466.00	2085.79	942.64	623.24	518.43
MTMR4					

* : not identified by published RNA-seq data (3).

** : Controls, reduction mammoplasty; TNBC, triple negative breast cancer primary tumor; ER+BC, ER positive brease cancer primary tumor; UBT_TNBC, uninvolved breast tissue adjacent to TNBC; UBT_ER+, uninvolved breast tissue adjacent to ER+BC.

1. **T. Souaiaia, Z. Frazier, T. Chen, ComB: SNP calling and mapping analysis for color and nucleotide space platforms. *Journal of computational biology : a journal of computational molecular cell biology* 18, 795 (Jun, 2011).**
2. **Y. Wang *et al.*, RseqFlow: workflows for RNA-Seq data analysis. *Bioinformatics* 27, 2598 (Sep 15, 2011).**
3. **K. E. Varley *et al.*, Recurrent read-through fusion transcripts in breast cancer. *Breast Cancer Res Treat* 146, 287 (Jul, 2014).**

Supplementary Sequence Information

REST-002 transcript variant differs in the 5' UTR compared to *REST-001*, but *REST-001* and *REST-002* variants encode the same REST protein. Therefore, we did not focus on non-coding RNA *REST-002*.

REST-001

Exon1-1 (57,774,075-57,774,379; 305 nt):

```
     GCAGCGTCCTGTGTTGGAATGTGCGGCTGCCGCGAGCTCGCGGCGCAGCAGCGGAGCGAG
     CGCCGCCGAGGCCCGGGGCCCCAGACCCTGGCGGCGGCTGCCGCAGCCGAGACGGCAGGG
     CGAGGCCCGGAGGCCTGAGCACCCTCTGCAGCCCCACTCCTGGGCCTTCTTGGTCCACGA
     CGGCCCCAGCACCCAACTTTACCACCCTCCCCCACCTCTCCCCCGAAACTCCAGCAACAA
 #1  AGAAAAGTAGTCGGAGAAGGAGCGGCGACTCAGGGTCGCCCGCCCCTCCTCACCGAGGAA   #9
     GGCCG
```

Exon2 (57,776,796-57,777,702: 907 nt):

```
             start codon            <-----------------------   A

 #9  AATACAGTTATGGCCACCCAGGTAATGGGGCAGTCTTCTGGAGGAGGAGGGCTGTTTACC (F REST-N)
     AGCAGTGGCAACATTGGAATGGCCCTGCCTAACGACATGTATGACTTGCATGACCTTTCC  si-REST_2 #1
     AAAGCTGAACTGGCCGCACCTCAGCTTATTATGCTGGCAAATGTGGCCTTAACTGGGGAA  (R REST-N)
     GTAAATGGCAGCTGCTGTGATTACCTGGTCGGTGAAGAAAGACAGATGGCAGAACTGATG
     CCGGTTGGGGATAACAACTTTTCAGATAGTGAAGAAGGAGAAGGACTTGAAGAGTCTGCT
  #5 GATATAAAAGGTGAACCTCATGGACTGGAAAACATGGAACTGAGAAGTTTGGAACTCAGC
     GTCGTAGAACCTCAGCCTGTATTTGAGGCATCAGGTGCTCCAGATATTTACAGTTCAAAT
     AAAGATCTTCCCCCTGAAACACCTGGAGCGGAGGACAAAGGCAAGAGCTCGAAGACCAAA
     CCCTTTCGCTGTAAGCCATGCCAATATGAAGCAGAATCTGAAGAACAGTTTGTGCATCAC
     ATCAGAGTTCACAGTGCTAAGAAATTTTTTGTGGAAGAGAGTGCAGAGAAGCAGGCAAAA(start R-004)
     GCCAGGGAATCTGGCTCTTCCACTGCAGAAGAGGGAGATTTCTCCAAGGGCCCCATTCGC
     TGTGACCGCTGCGGCTACAATACTAATCGATATGATCACTATACAGCACACCTGAAACAC
     CACACCAGAGCTGGGGATAATGAGCGAGTCTACAAGTGTATCATTTGCACATACACAACA
     GTGAGCGAGTATCACTGGAGGAAACATTTAAGAAACCATTTTCCAAGGAAAGTATACACA  (F REST new)
     TGTGGAAAATGCAACTATTTTTCAGACAGAAAAAACAATTATGTTCAGCATGTTAGAACT  (F REST-M)
     CATACAG
```

Exon3 (57,785,953-57,786,036: 84 nt):

```
FREST-M GAGAACGCCCATATAAATGTGAACTTTGTCCTTACTCAAGTTCTCAGAAGACTCATCTAA
        CTAGACATATGCGTACTCATTCAG                                      si-REST_1 #4
```

Exon4 (57,796,007- 57,802,010: 6004 nt):

```
      GTGAGAAGCCATTTAAATGTGATCAGTGCAGTTATGTGGCCTCTAATCAACATGAAGTAA
     CCCGCCATGCAAGACAGGTTCACAATGGGCCTAAACCTCTTAATTGCCCACACTGTGATT  si-REST_2 #2
     ACAAAACAGCAGATAGAAGCAACTTCAAAAAACATGTAGAGCTACATGTGAACCCACGGC  (R REST-M)
     AGTTC AATTGCCCTGTATGTGACTATGCAGCTTCCAAGAAGTGTAATCTACAGTATCACT (R-new REST)
     TCAAATCTAAGCATCCTACTTGTCCTAATAAAACAATGGATGTCTCAAAAGTGAAACTAA  si-REST_1 #3
     AGAAAACCAAAAAACGAGAGGCTGACTTGCCTGATAATATTACCAATGAAAAAACAGAAA
     TAGAACAAACAAAAATAAAAGGGGATGTGGCTGGAAAGAAAAATGAAAAGTCCGTCAAAG
     CAGAGAAAAGAGATGTCTCAAAAGAGAAAAAGCCTTCTAATAATGTGTCAGTGATCCAGG
     TGACTACCAGAACTCGAAAATCAGTAACAGAGGTGAAAGAGATGGATGTGCATACAGGAA
     GCAATTCAGAAAAATTCAGTAAAACTAAGAAAAGCAAAAGGAAGCTGGAAGTTGACAGCC
```

ATTCTTTACATGGTCCTGTGAATGATGAGGAATCTTCAACAAAAAAGAAAAAGAAGGTAG
AAAGCAAATCCAAAAATAATAGTCAGGAAGTGCCAAAGGGTGACAGCAAAGTGGAGGAGA
ATAAAAAGCAAAATACTTGCATGAAAAAAAGTACAAAGAAGAAAACTCTGAAAAATAAAT
CAAGTAAGAAAAGCAGTAAGCCTCCTCAGAAGGAACCTGTTGAGAAGGGATCTGCTCAGA
TGGACCCTCCTCAGATGGGGCCTGCTCCCACAGAGGCGGTTCAGAAGGGGCCCGTTCAGG
TGGAGCCGCCACCTCCCATGGAGCATGCTCAGATGGAGGGTGCCCAGATACGGCCTGCTC si-REST_2 #3
CTGACGAGCCTGTTCAGATGGAGGTGGTTCAGGAGGGGCCTGCTCAGAAGGAGCTGCTGC
CTCCCGTGGAGCCTGCTCAGATGGTGGGTGCCCAAATTGTACTTGCTCACATGGAGCTGC
CTCCTCCCATGGAGACTGCTCAGACGGAGGTTGCCCAAATGGGGCCTGCTCCCATGGAAC
CTGCTCAGATGGAGGTTGCCCAGGTAGAATCTGCTCCCATGCAGGTGGTCCAGAAGGAGC
CTGTTCAGATGGAGCTGTCTCCTCCCATGGAGGTGGTCCAGAAGGAGCCTGTTCAGATAG
AGCTGTCTCCTCCCATGGAGGTGGTCCAGAAGGAACCTGTTAAGATAGAGCTGTCTCCTC
CCATAGAGGTGGTCCAGAAGGAGCCTGTTCAGATGGAGTTGTCTCCTCCCATGGGGGTGG
TTCAGAAGGAGCCTGCTCAGAGGGAGCCACCTCCTCCCAGAGAGCCTCCCCTTCACATGG
AGCCAATTTCCAAAAAGCCTCCTCTCCGAAAAGATAAAAAGGAAAAGTCTAACATGCAGA
GTGAAAGGGCACGGAAGGAGCAAGTCCTTATTGAAGTTGGCTTAGTGCCTGTTAAAGATA
GCTGGCTTCTAAAGGAAAGTGTAAGCACAGAGGATCTCTCACCACCATCACCACCACTGC
CAAAGGAAAATTTAAGAGAAGAGGCATCAGGAGACCAAAAATTACTCAACACAGGTGAAG
GAAATAAAGAAGCCCCTCTTCAGAAAGTAGGAGCAGAAGAGGCAGATGAGAGCCTACCTG
GTCTTGCTGCTAATATCAACGAATCTACCCATATTTCATCCTCTGGACAAAACTTGAATA
CGCCAGAG**GGTGAAACTTTAAATGGTA**AACATCAGACTGACAGTATAGTTTGTGAAATGA si-REST_1 #1
si-REST_2 #4

AAATGGACACTGATCAGAACACAAGAGA**GAATCTCACTGGTATAAAT**TCAACAGTTGAAG si-REST_1 #2
AACCAGTTTCACCAATGCTTCCCCCTTCAGCAGTAGAAGAACGTGAAGCAGTGTCCAAAA (F REST-C)
CTGCACTGGCATCACCTCCTGCTACAATGGCAGCAAATGAGTCTCAGGAAATTGATGAAG (R REST-C)
ATGAAGGCATCCACAGCCATGAAGGAAGTGACCTAAGTGACAACATGTCAGAGGGTAGTG
ATGATTCTGGATTGCATGGGGCTCGGCCAGTTCCACAAGAATCTAGCAGAAAAAATGCAA
AGGAAGCCTTGGCAGTCAAAGCGGCTAAGGGAGATTTTGTTTGTATCTTCTGTGATCGTT
CTTTCAGAAAGGGAAAAGATTACAGCAAACACCTCAATCGCCATTTGGTTAATGTGTACT
ATCTTGAAGAAGCAGCTCAAGGGCAGGAG**TAA**TGAAACTTTGAACAAGGTTTCAGTTCTT
AGTTTGTAAGGTATATTACATTTTATATTCATTTATGATAGCAGACAACCTTTTAAGATT
GCTTTAATTAGTATCTGATGTTGATTTTTAAGTGGCATTCTTTTCCTTAGGACTTTTTAT
GTATACCTGTTGATTGTTGTGTAAATTTTAGTAAATCTAAGAGAGTGTACTAAACCAGCA
GGTATCTGTTAGCTTATGTGTTTAATTGAAATTAGAAGGCTAAGATGGTATAACAGCATT
TTATTGCTTTGTCCAGCTACAACTTGTCATTTTTTTCTCCATGTCTTATCTTCCTGTTTC
ACTTTAGTTTATTCTTCGTTTTTTATTGAGATCTATAAAAAATTGGCTTACTTAATAGCA
AATTACTTGAAGAATTTGCCTGCTTTATATAAAGTTAGCACTTTAAGATTTTTTTTTTTA
GAGATGAGAAGACATTTAAATTGAAGAAAAATTCCCCCAGCAATAGACAGTCTATCAGTC
CAAGTATTTACTTCCTGAGTTTTGATCAATATTTTTTATTTGTGTATGTTAATCGTCATA
AAAACAGTGATTTTGGTGTGTTTTTTATTTTGGTGCTTTAATGGCTTAAGATGTTGCACA
TTTTTTTTTTCTTTTGGTTTCTGTTTATGTTTTTTTGCCTATGCAGTTAAATTTTTCCTA
GAAATAGCATTTGTGTTGAACAGTAACACTTTATACATATATATATGCATGTTTATTTTG
TTTGGCGTCTTTGGAGGGATGCTTTTAGACTTGTTTGCAAAAGGGCAGTTTTCTTTTTCT
TTGCTGCAGTTGTCTATTTTGCAGAATAATAGTGTGTGCAAGTTTGTGAGCAAATGAAAT
ATGCAGGTTCAATCTATTGATTTTGATTTTTACATCTTATATCTATGCCAGAATCTGTAT
TTCATATAACTTATTTATTTCGAATGGATGTAGTAAATTCACAGCTATCAGTTTTGATTT
TGCAATAAATAAACCACTAGGTTGCATGTCGAACAAATTTTTATCTCAAATACCAACCAT
CAGTTTTTTTTTTCATGTGTTTTGGTACAGCTAATTCCTAATTGTAGAGTGTTAAATGTT
TGAGGAGAACCTTTTCTCATAGATGGTTGGTGTTCATATGGCTACTTTACAATAAAGAGA
ACTGTAAGTGATATTTGGAAACTACAAACCTGGAATTAGGAGATATAATTATTCCTTCAA
GTTTTATAGAATATCACTTGGGAGATTCCAAAGCCATAGCTATTACGCGGCAAACCTAGG
ATAAGAAAGGTAGTATGAGTGCTGGTAGACCAGCTGCAACTTTCCTATACAGTGAAAAAG
GCTGGTGAAACAAGTACAGTCCAGATTTTTTAAAATCATACTTTCTCAGGGATCTCCACA
AACTGGTGGGTGTCCTGGCTGTCTGTGTGATAGCCTCTTTCTATAGGTGAGGCCTCAAAT
GAATTGCAGCTATCCTGGTGTTCCTATGAGGGCACTTTGTATGAAAAAGGGCATGTACTC
CAAAACATTTTTGTAGGTTCTTTGGCCAGTTGCCAAAGAGTGTGAAAGAATCCAATAGAG
GATTTTTCTTACTGATAGCAGTCATTCATTGCAGTAAAATAAAATATGATCCCATTAGGG
AATCTTGAATTCTGACCTCCCATACTCCGTTTTGAAATAACCACTTTATATTTCATTTTT
TAAAAATCTGATGATCTCTTTGAGGCAGGTTTCAGATTTGGCAGTACAACATGAAAGATT
AGGAAAAGCATTAATAACGTGTGGGTGGAAAGCTTGTTAAAAATCTGAGAGTGAAGTTTG

AGTTAAAAGTTGTTTGAACATGGCATTGACTGGGAGGCCAAAGATTTAAAGAAGCGGAAG
ATTCTTCTCTTAAGACATGAGGAGTAAGTTGTGTGATAATGGTATGTGTTTTGTGTGCAT
GAATGGACATTGTAAATGTTGAATTCTAGGCTCCGACAATCATTGTCAACAGAAGATCAA
GCTGCAAATATTTATGTTTTAAAACTTAAATTATAAAGCTAGTTAAGTCTTTCTAATGAC
TAGTTTTAATGTTCATGGGTACATTTTACCTAAGTTACCGTTTACATTGTATAGAAAAAG
ATACATCTTAAGCACAGATTGGTTATTAGGAATTAGTTTGGGGAAGAGGTTTTTTTGTGG
ATTCTTTCATACTGCAAAGAAAAACCATTTGCCTTTTGGGGAATTGAGCTAACTTCTAAT
CTAGTCTTAAGACTAGAATGCTAAAAACAAAAACATGAAGGAAATTAAAACCCCTTATTA
TTAAATTGATTTGTAAAAACATTGTTACTGGAAATTTATTGGACTTGAGGCCTTCCTCCA
GAAAATAAGGACTTGATTGTCAGGCCTATATTAGGTTCTGAACCTTAATGCCATGTATTT
GTACTTACTAAAAATTGTTTCAATGAAAAGTACATTAGCAGTATGAACTTCTGGTCCAGT
TGGAAGTTTTTCCATTTGAAAAATGTGATGTTTGCATGGAACTGTTTGAAACTTTTTTAT
TTTCTAGTCCCCCTCCCCCACACTGGATAGAATTTAGCCTAGAATTTTCCCTTTGGATAA
AAGAACAAAAATTGAACATGTTATTTGTAAATTGATGTTTAGTAATTAGTGATAAACTTG
AAATACTAGCATATATTATAAGCCTTAATCTTAGGTAGTCTTATGAAAATGAATCTCTTA
ACTATCTTTTGAACCTGTATTCACATTGGTTTTCAAGATATTTTAAGTTATATTTTTTCC
TCTTTTCAGAGCTGCTTCTTATTCTGGGGCTACTTTTTTTTTTAGTTGTGTAATTCACAA
AGGGCTGCATTTTTTTTTTTTTTTAATAAGGCTTATAACTATGGCTGGATCTTTTGCTCT
AGTCTTCTAAGAAGGGCCATTTTATTTTTTAGAGTCACTTCTAAAGTCATGTGGTAATTA
ACTTTGGAGACTGTTTTGCGTATGAGTGCTGATACAAATTAAAACCCAAGTAGACCTCAT
TGCATGTCACCCTATGAATGTTGACAATGGAAGGAATACCTTGCCTGTAGTATACTGTCA
CTTCTGGATTGATAAGCTGAGGAAGAAAGTTAAGTTTCTTTTTTACATAAGTCAGAAAAA
CTTACAGCTGGTGTTCCTAGTTTCCTGGTTGACCTCAGCAGATGAAGTGAACAGATAGTG
TTAATTCAGATTGAAGAAATTATCTGAATCTTGGTTTGTGTAGATTTACAATCTACATGC
AATATTAACTAAATCAGATAGCTTTTACAGTTTCACATGTGTACATAGGTTCCCTCCCGG
TCCCTTCCATATCCATTAGTTATTGAACTTTCTAAACTGGCATTGAAACATTACAACAAT
GTTTTGTTGCACCAATTTTATAAACTTAAGCAGTGCAATACGTGTTACTTTTCTGAGGCA
AACCAAAGGTAAATTTCTCAAGGTTCTTGCTGCCTTCTTTAGCAGCATTTGATGGAAGAT
CTTTTATACATTTGTAATAGATAAAAATAAACCAGATTGCAAATCCTTTTTTAAAATCCT
AAACCATGTACCAAGTTTTTGGTCCAAATTATGTAGGATAAGTTAAACTTAAATTGCATT
CTATTAACCAATATGAGTGTATTTCTGTAAGCATAGTTATGTTGAAATAAAGTTTTAAAA
ACCA

Black font: Coding regions
Purple font: Untranslated regions (UTRs)

REST-004

***Exon2** (57,777,378-57,777,702: 325 nt):

*GCAGAGAAGCAGGCAAAA
GCCAGGGAATCTGGCTCTTCCACTGCAGAAGAGGGAGATTTCTCCAAGGGCCCCATTCGC
TGTGACCGCTGCGGCTACAATACTAATCGATATGATCACTATACAGCACACCTGAAACAC
#4 CACACCAGAGCTGGGGATAATGAGCGAGTCTACAAGTGTATCATTTGCACATACACAACA
GTGAGCGAGTATCACTGGAGGAAACATTTAAGAAACCATTTTCCAAGGAAAGTATACACA
TGTGGAAAATGCAACTATTTTTCAGACAGAAAAAACAATTATGTTCAGCATGTTAGAACT #6
CATACAG

Exon3 (57,785,953-57,786,036: 84nt):
#6 **GAGAACGCCCATATAAATG**TGAACTTTGTCCTTACTCAAGTTCTCAGAAGACTCATCTAA
CTAGA**CATATGCGTACTCATTCAG** R4

Exon N (57,793,781-57,793,830; 50 nt): *R-004 unique*
C ←-----------
R4 **TGGGGTATGGATACCATTTGGTAATATTTACTAGAGTGTGATCTAGATGG**

Dsi-R004**(IDT) : 5'-**GTATGGATACCATTTGGTAATAT**
si-*R004* (Thermo Scien.): 5'-**GTGTGATCTAGATGGGTGA**
si-*R004* (Ambion): 5'-**TGTGATCTAGATGGGTGAG**

Exon 4 (57,796,007-57,796,125; 119):
<-------- C
GTGAGAAGCCATTTAAATGTGATCAGTGCAGTTATGTGGCCTCTAATCAACATGAAGTAA
CCCGCCATGCAAGACAGGTTCACAATGGGCCTAAACCTCTTAATTGCCCACACTGTGAT

REST-003

Exon 1-3 (57,775,356-57,775,753: 398nt):
#3

G**AGTGTCGGGGCGACTCCCG**CGAGTTGGTGTGTAAATGTGTGCAGTGAGCGGGCCGAGGC
GAGAGGAAGCGAGAGCGCCCGAGTTT**GCAAAGAGCTGCTTTGTGTTTGGTA**TTTTGAGAA Dsi-*R003*
AATGGCCGAATG**CCTATTTCAGTTAGGAATGCC**GCCGCCGAGGCAGGCGCTGGTG**GCAGC** si-R003(Dha)
B ←-------------------
AGCACGGAGGAATTCGTGCAGCACGTTGGGCTTTCGGGCTGTGCCTGGACGGTCTTCCTA
ACAATCGTTAAAATTCCATTTGGGGTGGCAGTTAGGGGTTAGGGAGCAATTAGAAAA**GTC** **#8**
GATGTTGGGCCAAATTACCCAATAGCTTGGGCTGTTCAGCCCCGGGACAGCCTTCGCCCT
CTGTTTTCTGTGATGGCATTTGCTTCCAACTTTTGTTG

Exon2 (57,776,796-57,776,854:59 nt)

AATACAGTT**ATG**GCCACCCAGGTAATGGGGCAGT**CTTCTGGAGGAGGAGGGCTGTTTAC**
<------------------------ A

REST-002

Exon 1-2 (57,775,079-57,775,205: 127nt):

#2 CGGCGCTCGGAGCCCGACGCCTCGCGAGGGCGCCCGCGGAGCCTCCCCGGCCCTGGGCGT
TGGGCGAGCCCCGGGGCGGTCGGAGGGGCCCGGGGGCGGTCGGAGAAGCCCG**GACGCCGG**
CTGCGCG #7

Exon2 (57,776,796-57,777,181: 386 nt):

#7 **AATACAGTTATGGC**CACCCAGGTAATGGGGCAGT**CTTCTGGAGGAGGAGGGC**TGTTTACC
AGCAGTGGCAACATTGGAATGGCCCTGCCTAACGACATGTATGACTTGCATGACCTTTCC
AAAGCTGAACTGGCCGCACCTCAGCTTATTATGCTGGCAAATGTGGCCTTAACTGGGGAA
GTAAATGGCAGCTGCTGTGATTACCTGGTCGGTGAAGAAAGACAGATGGCAGAACTGATG
CCGGTTGGGGATAACAACTTTTCAGATAGTGAAGAAGGAGAAGGACTTGAAGAGTCTGCT
GATATAAAAGGTGAACCTCATGGACTGGAAAACATGGAACTGAGAAGTTTGGAACTCAGC
GTCGTAGAACCTCAGCCTGTATTTGA
